# The impact of a reproductive health voucher in Uganda using a quasi-experimental matching design

**DOI:** 10.1186/s12978-024-01812-2

**Published:** 2024-06-07

**Authors:** Christian Andersson, Tonny Kawuki, Jonas Månsson, Christine Nankaja, Krister Sund, Emma Wigren, Mathias Mulumba Zungu

**Affiliations:** 1 The Swedish National Audit Office, Stockholm, 102 33 Sweden; 2 Blekinge Institute of Technology, Karlskrona, 371 41 Sweden; 3 The Office of the Auditor General Uganda, Kampala, Uganda; 4 The Swedish Institute for Social Research, Stockholm, 106 91 Sweden

**Keywords:** Voucher, Infant mortality, Maternity services, Matching, Quasi-experimental trial, Uganda, I12, I38, N37

## Abstract

This study assesses the impact of a voucher project that targeted vulnerable and poor pregnant women in Uganda. Highly subsidised vouchers gave access to a package of safe delivery services consisting of four antenatal visits, safe delivery, one postnatal visit, the treatment and management of selected pregnancy-related medical conditions and complications, and emergency transport. Vouchers were sold during the project’s operational period from 2016 to 2019. This study covers 8 out of 25 project-benefiting districts in Uganda and a total of 1,881 pregnancies, including both beneficiary and non-beneficiary mothers. Using a matching design, the results show a positive effect on the survival of new-born babies. The difference in the survival rate between the control group and the treatment group is 5.4% points, indicating that the voucher project reduced infant mortality by more than 65 per cent.

## Introduction

During recent decades, the number of deaths of children under 5 years of age fell globally from 12.7 million in 1990 to 6.3 million in 2013, according to the World Health Organization WHO, [31]. Tremendous progress has been made, and the global under-5 mortality rate in 2018 was 39 deaths per 1,000 live births compared with 93 deaths per 1,000 live births in 1990 (United Nations Inter-Agency Group for Child Mortality Estimation UN IGME [23]. Several initiatives have been instigated to decrease both child and maternal mortality over the years. For example, the Millennium Development Goals (MDGs) targeted the reduction of the under-5 mortality rate by two-thirds between 1990 and 2015 and at the same time aimed to reduce the maternal mortality ratio by three-quarters. The reduction of child mortality is also reflected in several of the United Nations’ Sustainable Development Goals (SDGs). For example, one target is to end preventable deaths of new-borns and children under 5 years old by 2030. Furthermore, all countries should aim to reduce neonatal mortality to at least as low as 12 deaths per 1,000 live births and under-5 mortality to at least as low as 25 deaths per 1,000 live births.

More than 98 per cent of neonatal deaths occur in low- and middle-income countries. Sub-Saharan Africa is the region in the world with the highest level of child and neonatal mortality. In this region, 1 in 9 children dies before turning 5 years old, a rate that is 18 times higher than the average for developed regions [20]. Beyond child mortality complications during pregnancy, childbirth and the postnatal period are the leading causes of death and disability among women of reproductive age in developing countries. According to the WHO [30], almost 300,000 women died during or following pregnancy and childbirth in 2017. Almost all these deaths (94 per cent) occurred in low-resource settings, and most could have been prevented.

In Uganda, the government is striving to reduce both child and maternal mortality. The progress has been slow and uneven over the years. Uganda was not able to achieve the MDGs within these areas Republic of Uganda [17]. In the country, 17.5 per cent of all female deaths were pregnancy related. In addition, the infant mortality rate was 34 deaths per 1,000 live births and the neonatal mortality rate was 20 deaths per 1,000 live births in 2018 UN IGME [11, 23] show even higher neonatal mortality (27 deaths per 1,000 live births) in rural communities in Eastern Uganda and that home visits by community health workers and antenatal visits can lower the risk of neonatal death. A possible explanation for the high mortality rate is that institutional delivery remains low in rural areas and the delivery rate at health facilities was estimated to be only 52 per cent in 2011 Uganda Bureau of Statistics [21]. Additionally, according to the World Bank [29] a significant number of women deliver their babies at home with assistance of unskilled birth attendants, such as traditional birth attendants or relatives, or without any support at all. The main reasons for women not delivering at a health facility are: financial limitations, long distances to health facilities and a preference for traditional child birth positions [3, 26]. The WHO [30] indicates that skilled care before, during and after childbirth can save the lives of both women and new-borns, further stating that: ‘It is particularly important that all births are attended by skilled health professionals, as timely management and treatment can make the difference between life and death for the mother as well as for the baby’. The World Bank [29] also indicates that maternal care is impeded by a persistently high fertility rate (approximately 5.7 children per woman) in Uganda.

To increase access to skilled care, the Ministry of Health established the Uganda Reproductive Health Voucher Project (URHVP). This demand-side financing (also called output-based aid and discussed in more detail in Sect. 4) project was expected to enhance access to quality obstetric care among rural and poor pregnant women as a way of providing safe delivery services. This would contribute to the government’s goal to decrease both maternal and child mortality. The project focused on poor women in Uganda who face challenges in accessing safe delivery services. Most of the targeted women reside in rural areas, where safe delivery services in general are inadequate and hard to access (Office of the Auditor General Uganda [16]. The maternal services offered by public health facilities are meant to be free see, for example, Deininger and Mpuga [6], but, owing to shortages of drugs and supplies, the patients are usually required to buy various commodities, which the targeted women are not able to do due to financial constraints. Because of this, the targeting was expected to reduce the financial barriers and of course promote the importance of accessing services that reduce the risks associated with pregnancy and childbirth Office of the Auditor General Uganda [16].

Previous research argues that there is a need for further knowledge regarding these kinds of voucher systems, especially research that can establish the impact of voucher projects and measure different health outcomes. Overall, there is little evidence in the literature that documents the causal effect of voucher programmes. No previous studies evaluate the effect of voucher programmes for pregnant and vulnerable mothers in Uganda using health outcomes and modern econometric methods. However, there are some evidence from Uganda that the use of vouchers for pregnant women can increase health facility utilisation [7, 12]. In this study, unique and very detailed individual data were collected to isolate the impact of the project. The purpose was to establish the causal impact of a reproductive health voucher in Uganda on child mortality using a quasi-experimental matching design.

The paper is organized as follows. Section 2 provides the brief background of the voucher project. Section 3 present previous research and Sect. 4 contains the theoretical framework and the empirical strategy. Section 5 examines the data, and results are reported in Sect. 6. Finally, Sect. 7 concludes and discusses policy implications.

## Background

To increase access to skilled care, the Ministry of Health established the Uganda Reproductive Health Voucher Project (URHVP), which was expected to enhance access to quality obstetric care among rural and poor pregnant women. The Swedish International Development Agency (SIDA), through the Global Partnership on Output Based Aid, extended a grant of USD 13.3 million to the Government of Uganda to deliver the URHVP. The project was approved in 2014 and closed in 2019 World Bank [28].

To implement the voucher programme, the Ministry of Health contracted the voucher management agency Marie Stopes Uganda (MSU) to serve as the project implementation agency. MSU has supported the Government of Uganda in the development of policies and guidelines related to family planning and reproductive health services for several years. In the URHVP, the project team from MSU comprised a project manager, who was the team leader, as well as a health specialist, a monitoring and evaluation specialist, a financial management and administration specialist and a communications specialist, all of whom reported to the project manager. MSU, in turn, contracted community-based distributors that sold and distributed the vouchers to pregnant women within the defined catchment areas. The pregnant women purchased vouchers at a subsidized price of UGX 4,000, approximately USD 1.1 (Office of the Auditor General Uganda [16]).

The URHVP was initially expected to deliver skilled attendance to support 132,400 pregnant women through the subsidized voucher scheme, offering a package of safe delivery services consisting of four antenatal visits, safe delivery, one postnatal visit, the treatment and management of selected pregnancy-related medical conditions and complications (including caesarean sections) and emergency transport.^1^ This target was revised mid-term to cover 156,400 mothers World Bank [28].

The URHVP was implemented in 12 districts of western Uganda and 13 districts of eastern Uganda.^2^ Following the selection of the target sub-counties, the Voucher Management Agent deployed BCCs to undertake mass enrollment of pregnant women in the localities. They then offered the coupons to the qualified ladies for a value of UGX 4,000 and instructed them on how to benefit from the project’s items. The primary beneficiaries were poor and vulnerable pregnant women residing within the catchment areas of the contracted health facilities. The contracted voucher service providers and surrounding communities were the secondary beneficiaries.

To identify poor and vulnerable pregnant women the URHVP used a customized poverty grading tool developed by the World Bank [1] to select eligible beneficiaries. To establish eligibility, the project staff subjected potential mothers to the customized poverty grading tool and scored them against selected indicators to assess their level of poverty see World Bank [28]. Mothers who scored 0–9 marks were assessed as poor; those who scored 10–15 marks were assessed as medium; and those scoring above 15 marks were assessed as rich. Pregnant women who scored 12 or lower on the poverty grading tool were eligible to buy the vouchers. This rule was implemented very strictly, and in only a few cases was it observed that pregnant women who scored higher than 12 on the test were able to buy the vouchers.

In 2017, the World Bank [28] conducted a mid-term review of the project and noted some challenges that were likely to hinder the achievement of the project’s outcomes and impacts. First, the project’s commencement was delayed by 18 months. This could affect the project’s outcomes if, for example, the costs of the service increased more than the appreciation of the United States dollar. Second, the report encouraged the voucher management agency to strengthen its information, education and communication activities to counter project beneficiaries’ low level of attendance at the fourth antenatal care visit and postnatal care. They noted that there was low awareness of the complete package of health services attached to the voucher, which could result in the failure to maximize the outcome of access to skilled care.

The report also noted that there were 30 providers of the service in Western Uganda compared with nine in Eastern Uganda. Additionally, in some districts, the ambulance services were insufficient, leading to reliance on various modes of ad hoc transport arrangements. The findings in the review noted especially the poor management of referrals in Eastern Uganda and concluded that there was a risk of failure to achieve the project’s outcome related to the handling of pregnancy complications in this part of the country.

Finally, the report noted that, in similar projects supporting maternal health in the country, there is a risk that the changes observed in pregnant mothers’ access to skilled care might be attributable not to the voucher project but to other similar interventions by the government. The World Bank therefore argued that an impact assessment would be necessary to isolate the effect of the project from the many changes observed in pregnant mothers’ access to skilled care and other desired outcomes in the delivery of maternal health services.

## Previous research

Demand-side financing or output-based aid has been on the agenda for many years in regard to providing low-income populations with reproductive health services. Bhatia and Gorter [4] provide a good background to the concepts of output-based aid and to the research within the area up to 2007. The authors argue that this type of aid can increase access to reproductive and child health services. They also assert that demand-side financing not only promotes equity through improved access and better targeting of subsidies but also provides incentives for efficiency and provider choice by involving the private sector. US AID [24] states that there is a clear need for rigorous research that can conclusively establish the impact of voucher programmes. Ensor [8] show that demand side financing using vouchers has a potential to provide more targeted services to the poor.

Meyer Brody et al. [14] present a review of the literature evaluating the impact of voucher programmes on the use and quality of health goods and services in developing countries. The findings suggest that there is evidence that health voucher programmes have been successful in increasing the utilization of health services. There is also modest evidence that voucher programmes can target specific populations effectively and improve the quality of services.

Eva et al. [9] review 24 different voucher programmes for family planning and sexual and reproductive health across 11 countries in Africa and Asia between 2005 and 2015. All the reviewed programmes were managed by Marie Stopes International (MSI)^3^. Three of these programmes were implemented in Uganda. The outcome measures in the reviewed studies were the uptake of services, service use among specific subgroups, users’ satisfaction with the service quality and the efficiency of service delivery. The overall results show that the service uptake increased following the implementation and that most programmes were successful in reaching subgroups such as poor people. The programmes also showed high user satisfaction, but the results concerning efficiency were mixed. For the programmes in Uganda, the results showed an increase in the proportion of health facility deliveries, and voucher users were found to be more likely to use health facilities for delivery than to deliver at home. The user satisfaction in the Ugandan programmes was found to be high; 94 per cent of voucher users were satisfied with the services compared with 76 per cent of non-voucher users. The authors conclude that the programmes successfully increased the service uptake and that they were effective in reaching the poor when a poverty grading tool was used to assess potential users and limit eligibility. However, they also point to some key areas for future research. Most importantly, none of the reviewed programmes evaluated the long-term effects or focused on the health outcomes of voucher users.

The relationship between health facility delivery and neonatal mortality was reviewed by [20] using a meta-analysis. Studying the results from 19 studies that fulfilled the inclusion restriction, they found that health facility delivery reduces the risk of neonatal mortality by 29 per cent in low- and middle-income countries. However, this study does not consider voucher programmes to increase health facility delivery.

Very few of the previous studies use modern study designs that would enable them to draw conclusions about the causal effect of voucher programmes. One exception is Keya et al. [13], who follow a difference-in-difference approach to evaluate a voucher programme aimed at delivering care in Bangladesh. The results show a significant increase in public health facility use and an increase in delivery complication management care. The study, however, does not consider the health outcomes of the voucher programme.

Several studies focus on voucher programmes in Uganda, evaluating different outcomes [7] study access to institutional deliveries using both demand and supply side incentives in two districts in Eastern Uganda. The authors show that the use of vouchers given to pregnant women for antenatal, delivery and postnatal care as well as for transportation increased the number of safe deliveries in the intervention area.

Kanya et al. [12] show that a voucher programme that subsidized four antenatal care visits, delivery and post-natal care services for economically disadvantaged women in South-western Uganda had a potential to increase facility-based births among poor women. The also show a negative correlation between the poverty density in a district and the proportion of births that were covered by the programme, implying a need to improve voucher coverage in districts with high poverty levels.

Obare et al. [15] present results from a quasi-experimental evaluation of the Uganda reproductive health voucher programme, considering the following outcomes: knowledge, behaviour, quality and out-of-pocket spending. They find a 16-percentage point increase in private facility deliveries and a decrease in home deliveries for voucher users. They conclude that the project is likely to have contributed to increasing private facility births in villages with voucher clients. The authors also point out the need for future research to link service uptake with health outcomes.

Alfonso et al. [2] study the cost-effectiveness of a voucher scheme combined with health system strengthening in rural Uganda using a difference-in-difference approach. The results show that the demand for births at a health facility for voucher users increased by 52.3% points. Using cost data and assumptions about the number of deaths averted, the study shows that the project was cost-efficient even under extreme assumptions.

## Theoretical framework

To measure the impact of the URHVP, two groups need to be identified: a treated group and a group that is not treated. In our case the treated group (beneficiaries) are women that scored 12 or lower on the poverty grading tool and decided to buy a voucher. The non-treated group (non-beneficiaries) consist of women that did not obtain a voucher. If the assignment to participation is made randomly, the evaluation problem consists of comparing the outcomes of the two groups to determine the impact of participation. However, in cases in which the assignment to a programme is not random, a potential problem can occur. If individuals are selected into a programme based on their characteristics, the difference in outcome can contain not only the programme effect but also the effect of being selected, that is, selection bias. Thus, the difference in observed outcomes can be divided into two components:

### Difference in outcome between treated and untreated = programme effect + selection effect

The selection effect can be both positive and negative. For example, if selected persons have characteristics that make them more likely to succeed, in our case more likely to give birth to a child that is alive, the difference in observed outcomes will be an overestimate of the true programme effect. On the other hand, if mothers have characteristics that make it more likely that they, or their children, will have severe medical concerns during or after birth are selected, the observed difference in outcomes will underestimate the true programme effect. In our case, vouchers are given to women who are among the most vulnerable, so just comparing the outcomes will most likely result in a downward bias of the true programme effect.

All types of quasi-experimental methods have advantages and disadvantages; however, common to all methods that use observed characteristics is that all the important variables that affect the selection to participate and the expected outcome need to be observed. This is called the conditional independence assumption (CIA) and requires discussion. If the CIA does not hold, we will still have selection bias, regardless of the method chosen.

In this study, we choose to use a propensity score matching (PSM) approach based on the work by Rubin [19] and Rosenbaum [18].^4^ The idea of PSM is to allow the data to mimic the selection that takes place by modelling the process. We motivate the methodological choice by the fact that the population under study is fairly homogeneous, and we have good control over the individual characteristics that are used to determine programme participation. Thus, our data and our knowledge, mainly based on interviews, give us the possibility to fulfil the conditions set out in the CIA. By using the poverty score and other observed characteristics, we first estimate the probability of being selected for both beneficiaries and non-beneficiaries. The novelty of the PSM method is that, after computing the predicted value to be assigned, this number captures all the variables, which makes it easy to match. After matching using the propensity scores, we end up with two groups, one treated and one untreated, with the same predicted probability of being assigned treatment, which would also be the case in a randomized control trial. After conducting the matching procedure, the programme’s impact can be estimated by comparing the average outcome of the treatment, or enrolled, group with the average outcome among a statistically matched subgroup of women who did not receive treatment.

## Data

The data used in this study come from a unique and very detailed dataset collected in the field by professional data collectors using a digitalized questionnaire. The data are individual micro data that provide us with a large number of individual characteristics, which will be necessary to carry out our econometric matching strategy and to make a causal interpretation of our results. The data were collected in October and November 2019.

The sampling was performed using the multistage stratified sampling strategy. The population of 25 project-implementing districts was grouped into 2 strata, that is, the eastern region (13 districts) and the western region (12 districts) of Uganda. A sample of 4 districts was randomly selected from both the eastern and western regions, and 10 villages were then selected from each district. In the last stage of the sampling, 20 mothers (10 beneficiaries and 10 non-beneficiaries) were chosen within each selected village. Over the period of the project, 10 beneficiaries were systematically selected. Equally, 10 non-beneficiaries were selected randomly within each of the 10 selected villages. The theoretical sample size necessary to be able to draw statistical inference was estimated to be around 1,600 mothers. These mothers were asked about all their pregnancies during the project period.

The data collection team, with support from the Uganda Bureau of Statistics (UBOS), then collected quantitative data on characteristics of interest from beneficiaries and non-beneficiaries in the sampled districts. This involved a detailed questionnaire, which was used to interview the mothers and capture the required information. The response rate of the survey was 92 per cent: 1,486 out of the expected sample of 1,600. Some of the interviewed women did not give birth within the studied time period between 2016 and 2019, making the total number of mothers used in our estimations 1,418. Of these interviewed women, 432 had multiple pregnancies during the phase of the project between 2016 and 2019. Since infant mortality is our outcome variable, we considered all these pregnancies, which could be with or without a voucher under the project.^5^ The final sample of pregnancies that was used in this study consisted of 1,881 pregnancies over the studied time period. In Tables 1 descriptive statistics are presented regarding pregnancies divided between the regions along with an indication of whether the mother used a voucher.

**Table 1:** Number of pregnancies during the studied time period for beneficiaries and non-beneficiaries in the sample

	Number of pregnancies for beneficiaries	Number of pregnancies for non-beneficiaries	Total
**Western region**	426	615	1,041
**Eastern region**	272	568	840
**Total**	698	1,183	1,881

As seen from Table 1, the data collection team managed to interview a somewhat higher number of women in the western region and therefore the number of pregnancies is higher in this region. The reason for the lower number of observations in the eastern region was the difficulty of identifying beneficiaries in the selected villages. This might be because of the poor implementation of the project in this region, as the World Bank indicates in its mid-term review World Bank [28].

Infant mortality is used as the outcome variable of interest. A discussion about problems with, for example, measurement errors can be found in US AID [25]: ‘There are two principal categories of estimation methods for calculating infant and child mortality rates: direct and indirect. Direct methods of calculation use data on the date of birth of children, their survival status, and the dates of death or ages at death of deceased children. Indirect methods use information on the survival status of children to specific age cohorts of mothers. The direct methods require data that are usually obtained only in specifically designed surveys with birth histories or from vital statistics systems. The estimation of infant mortality, using direct methods, depends on the correct reporting of age at death as under or over one year. The heaping of deaths at age 12 months is common, and to the extent that it causes a transfer of deaths across the one-year boundary, infant mortality rates may be somewhat underestimated.

### Descriptive statistics

The data for the assessment related to the project implementation period from January 2016 to 2019. This applied to mothers who purchased the voucher and used it.

Descriptive statistics of the sample are presented in Table 2 below. From the table, we can see that the groups are similar in many respects. However, the poverty score is higher, and the monthly expenditures are lower among the treated, as expected. In addition, fewer of the beneficiaries are married, they are slightly older, and they have somewhat more children than the non-beneficiaries.

**Table 2:** Characteristics of the survey respondents, mean values and standard deviations. Standard deviations within parentheses

	Beneficiaries	Non-beneficiaries
Share of live births	0.97 (0.167)	0.93 (0.26)
Number of children	2.87 (1.64)	2.57 (1.60)
Number of pregnancies	2.88 (1.62)	2.52 (1.45)
Age	28.16 (5.98)	26.64 (5.83)
Monthly expenditure, UGX	114,750 (188.83)	138,638 (246.16)
Poverty score	10.34 (1.72)	10.11 (1.58)
Share who are married	0.91 (0.28)	0.89 (0.31)
**Education**		
None	0.40 (0.49)	0.44 (0.50)
Primary	0.40 (0.49)	0.39 (0.49)
Lower-secondary schooling (ordinary level)	0.15 (0.36)	0.14 (0.34)
Upper-secondary schooling (advanced level)	0.04 (0.19)	0.03 (0.16)
**Religion**		
Roman Catholic	0.31 (0.46)	0.31 (0.46)
Islam	0.09 (0.29)	0.14 (0.34)
Anglican/Protestant	0.49 (0.50)	0.44 (0.50)
Pentecostal/Born Again/ Evangelical	0.09 (0.28)	0.10 (0.31)
Others	0.02 (0.13)	0.02 (0.12)
**Number of observations**	698	1,183

### Matching quality

**Fig. 1:**
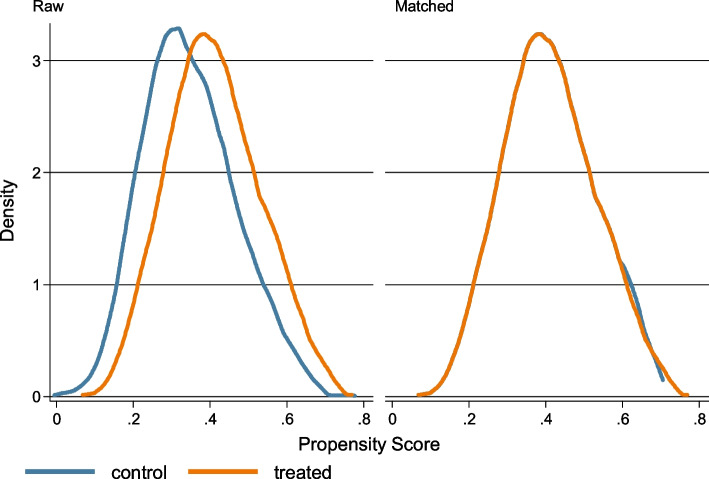
Kernel density plots of the estimated propensity scores for the control and treated groups

Figure 1 below shows the density plots before and after matching the sample on propensity scores.^6^ We observe no difference in the mean propensity scores and their distribution in the two groups after kernel matching, indicating that we were successful in our matching.^7^

From the results, we can see that almost all observations are on common support. Only seven observations are off support. Table 3 further explores the matching quality by reporting the standardized differences between the raw and matched samples. The standardized differences in the matched sample should be close to zero and the variance ratio should be close to one for a satisfactory matching quality.

**Table 3:** Covariance balance summary

	Standardized differences		Variance ratio	
	Raw	Matched	Raw	Matched
**Number of pregnancies**	0.24	0.00	1.25	0.95
**Mother’s age**	0.26	0.00	1.05	1.01
**Monthly expenditure, UGX**	-0.11	0.03	0.59	1.80
**Poverty score**	0.14	0.00	1.19	1.19
**Marital status (ref. married)**				
Divorced/separated	-0.08	0.01	0.80	1.04
**Level of education (ref. no education)**				
Primary schooling	0.02	0.00	1.01	1.00
Lower-secondary schooling (O-level)	0.05	-0.02	1.10	0.96
Upper-secondary schooling (A-level)	0.07	-0.01	1.41	0.97
**Religion (ref. Roman Catholic)**				
Islam	-0.14	0.02	0.70	1.07
Anglican/Protestant	0.11	-0.02	1.02	1.00
Pentecostal/Born Again/Evangelical	-0.06	0.01	0.85	1.03
Others	0.02	0.00	1.13	1.00
**Distance to nearest public hospital (ref. ≤ 1 km)**				
1–2 km	-0.14	-0.02	0.82	0.97
2–5 km	0.13	0.01	1.13	1.01
5–10 km	0.05	0.00	1.12	1.00
>10 km	0.07	0.02	1.32	1.09
**Year of pregnancy (ref. 2016)**				
2017	0.24	0.04	1.31	1.04
2018	0.,03	-0.03	1.02	0.98
2019	-0.22	0.01	0.62	1.04

For most of our variables, included in the first stage Probit estimation, the differences between the two groups are reduced. Overall, the differences from zero in means and the differences from one in variances are small, indicating that the matching on propensity scores has reduced the initial bias to a large extent.

## Results

### Is the intended treated group actually treated?

One problem with all projects in which the target population receives support for a certain action is dead weight loss (DWL). DWL is defined as the situation in which the target population would have chosen to be treated even without financial support. In this study, this would imply that pregnant women would have attended a health clinic regardless of the support given. The DWL could potentially be affected by the eligibility criteria for the voucher. The criteria to be eligible for the voucher scheme was that a pregnant woman scored 12 or lower on the poverty grading tool used. To obtain some information about DWL, a survey question was formulated as follows: ‘Would you have visited a health care clinic regardless of the support?’ Of the beneficiaries, 86 per cent indicated that they would still have attended the health facility to access services during their pregnancy regardless of whether they had a voucher. The corresponding number for the non-beneficiary group is very similar, 83 per cent. This result has two implications. Firstly, the voucher programme, for the majority of mothers, mainly represented a transfer from private consumption to public consumption. Secondly, the targeted group for the support, pregnant women who, due to financial restrictions, could not have attended health care clinics, were not reached to a large extent. The latter result could be either due to the project failing to identify the target population with high precision or the target group being too broadly defined. In fact, retrospective analysis of the poverty score shows that, overall, only 32 per cent of the beneficiaries were poor, i.e. scoring 8 or lower on the poverty grading tool, while the remaining 68 per cent were classified as medium or higher on the poverty scale. The eastern region was more affected as only 29 per cent of the selected beneficiaries were deemed poor according to the poverty grading tool compared to 33 per cent in the western region. The targeting of pregnant women who were not poor opposes the very objective of the project and consequently reduces its impact. It is also apparent that about 4 per cent of the project beneficiaries were ineligible. These had scored over 12 marks and yet they benefitted from the project. This is because, in some cases, poverty assessment was not undertaken at the mothers’ homes although many of the poverty parameters considered could only be assessed there, such as the mothers’ sanitation facilities, water source and shelter. Interviews with the mothers revealed that, when the vouchers were scarce, some mothers looked for the community-based distributors and purchased the voucher without proper poverty assessment on their premises, rendering the assessment ineffective.

To summarize, the finding is that the targeted group of pregnant and vulnerable women who, due to financial restrictions, could not afford to visit a health care clinic – could have been reached to a larger extent. This could for example have been achieved by having a lower eligibility level on the poverty grading tool. Since vouchers were scarce in many regions a better targeting could possibly have been achieved by only making pregnant women that scored 8 or lower on the poverty grading tool eligible for the voucher scheme. The fact that a higher level on the poverty grading tool was chosen for eligibility have most likely had an influence the impact of the project. Especially so since there is a non-ignorable number of treatment group members (voucher) who, in the absence of a voucher, still would have attended a health care clinic. The result reported in the following section should therefore be viewed as a lower limit regarding the impact size.

### Treatment effect on the treated

The objective of this study was to assess the impact of using a voucher on the beneficiaries of the URHVP. The outcome measure used was the survival of babies during pregnancy and birth by estimating the average difference in the probability of survival between beneficiaries and non-beneficiaries. To measure the effect of the voucher project, we made use of very rich and detailed data from a survey collected especially for this purpose. We applied a matching strategy to compare beneficiaries with non-beneficiaries and to estimate the effect of using the voucher.

A brief look at the raw data on the outcome indicator, namely the survival of the baby during pregnancy and birth, shows that 97 per cent of the babies of beneficiaries survived compared with 93 per cent of similar non-beneficiaries.^8^ This preliminary analysis indicates that beneficiaries were better off since their babies had a higher chance of surviving. However, these groups could differ in various ways that could influence their participation in the voucher programme. To estimate the effect, we applied our matching strategy described above. We performed a PSM that matched the beneficiary group with the non-beneficiary group based on women’s poverty score and the following variables: age, educational level, religious denomination, marital status, number of pregnancies, district, average household expenditure and year of pregnancy. The result from this main analysis is presented in Table 4 below.

**Table 4:** Effect of having participated in the voucher programme, ATT

Effect of participating in the voucher project on infant mortality	Number of observations
0.054^a^ (0.014)	1,881

^a^refers to statistical significance at the 1 per cent level.

The result shows a positive impact of the voucher project, with an average treatment effect of 0.054, which means that babies of the beneficiaries have a 5.4% point higher probability of survival during pregnancy and birth. The infant mortality rate for non-beneficiaries in the target group of poor and vulnerable women is 8.2 per cent. A reduction of 5.4% points means that, due to the project, the infant mortality rate in the targeted group fell to less than 3 per cent, that is, a reduction of 65 per cent. This also means that the infant mortality rate in the targeted group was somewhat lower than the average infant mortality rate in Uganda, which is 3.4 per cent.

### Differences in outcomes between the western and eastern regions

Interviews held with the project management at the Ministry of Health (MoH) and MSU showed that the eastern region had been identified at the mid-term review as having a lower likelihood of obtaining good outcomes than the western region. For example, the mid-term review mission indicated that there was a lower uptake of vouchers in the eastern region and fewer health units providing comprehensive obstetric care. It was also noted that the western region had a previous voucher scheme called *Child Plus*, dealing with family planning issues, which might have improved the chances of good implementation of the studied voucher scheme. However, interviews with MSU indicated that the structure and numbers of the project staff within the two regional offices were the same. In addition, the work plans provided showed the same allocation of resources (time, manpower and money) for monitoring and evaluation, training and mentorship for both regions. As noted earlier, this is also mentioned by the World Bank in a mid-term review. The review notes especially the poor management of referrals in Eastern Uganda, and the conclusion is that there was a risk of not achieving the project outcome related to the handling of pregnancy complications in the eastern region.

Despite this prior knowledge, no measures were placed within the design of the project to avert this imbalance, which makes it even more interesting to estimate the effect of the voucher project separately for the western and eastern regions. The result from this exercise is presented in Table 5.

**Table 5:** Effect of having participated in the voucher programme for the different regions, ATT. Standard errors within parentheses

	Effect of participating in the voucher project on infant mortality	Number of observations
Western region	0.076^a^ (0.016)	1,041
Eastern region	0.018 (0.017)	840

^a^refers to statistical significance at the 1 per cent level

A comparison of the impacts of the project in the western and eastern districts indicates that babies of beneficiaries in the western region have a 7.6% point higher probability of survival during pregnancy and birth than those of non-beneficiaries. However, for the eastern region, there is no evidence of a significant difference in the probability of survival of the babies of beneficiaries and non-beneficiaries.

It is likely that the indicated problems in the implementation of the project in the eastern region influenced the efficiency of the voucher scheme. It is also worth noting the strong and significant positive effect of almost 8 per cent in the western region, suggesting a large difference in the possibility of a child surviving pregnancy and delivery if the mother was a beneficiary of the studied voucher project.

### Sensitivity analysis

A sensitivity analysis was conducted to establish the stability of the estimated effect of the voucher project. To determine whether our results are stable, we estimated the effect on infant mortality using only the first pregnancy during the studied period between 2016 and 2019. This eliminated the risk that some mothers who gave birth multiple times during the studied time frame influenced the results. However, we could only include 1,418 births instead of the 1,881 in our complete sample. The result of this sensitivity analysis is provided in Table 6.

**Table 6:** Effect of having participated in the voucher programme: only the first birth in the studied period 2016–2019, ATT

Effect of participating in the voucher project on infant mortality	Number of observations
0.035^a^ (0.012)	1,418

^a^indicates statistical significance at the 1 per cent level

The conclusion from Table 7 is that there is still a positive and statistically significant effect on the survival of babies born to mothers who participated in the voucher project. The probability of survival is 3.5% points higher for babies of participating mothers than for babies of non-participating mothers.

We also estimated several different selection models. In the first basic model (Model 1), we only included the poverty score to match beneficiaries and non-beneficiaries. The logic is that much of information about the mothers is given by the poverty rating score.

We then increased the number of control variables to observe whether our estimated effect, that is, the difference in the survival of babies between beneficiaries and non-beneficiaries, changes when different variables are added. The results of this analysis are presented in Table 7.^9^

**Table 7:** Effect of having participated in the voucher programme on infant mortality. Different model specifications, ATT. Standard errors within parentheses

Variables	Model 1 (Basic model)	Model 2	Model 3	Model 4	Model 5
**Treatment effect**	0.048^a^ (0.012)	0.038^a^ (0.010)	0.046^a^ (0.012)	0.055^a^ (0.012)	0.054^a^ (0.014)
**Poverty score**	•	•	•	•	•
**Age**		•	•	•	•
**Level of education**		•	•	•	•
**Marital status**			•	•	•
**Average household expenditure**				•	•
**Religious denomination**					•
**Number of pregnancies**					•
**District of the respondents**					•
**Year of pregnancy**					•

^a^indicates statistical significance at the 1 per cent level

As can be seen from Table 7, the results are positive and significant in all the estimated models. The estimated survival rate of babies of beneficiaries is between 4 per cent and 7 per cent higher than that of babies of comparable mothers who did not use a voucher. It can, for example, be noted that the estimated effect of using a voucher on the survival of the child when we only control for the poverty grading score is around 5 per cent and statistically significant at the 1 per cent level. This is a very similar result to our main result, presented in Table 5. It is likely that the poverty score captures the differences between beneficiaries and non-beneficiaries to a large extent and that additional variables do not add much information. The main point is that our results are very stable and show a positive and statistically significant effect for all the estimated models.

We can also compare the result from the matched analysis with the estimated effect from a crude Probit model without matching but with the same explanatory variables. The Probit model produces an estimate of the treatment of 0.044 with a standard error of 0.01. This is similar to the result in the first columns in Table 7 above. The matched result changes from the crude estimate, albeit not by much, as household expenditures are introduced into the selection model. This is reassuring since large differences would indicate that selection might have been present in the distribution of the vouchers. If the vouchers had been randomly distributed, the crude Probit and the matched results would have been similar, which we are not too far from now.

### Cost efficiency of the voucher project

According to UNICEF, 23 per cent of children in Uganda live in households that are below the poverty line [22]. In this section, we present a rough calculation of the cost efficiency of introducing a programme such as the studied voucher project nationwide in Uganda. Such a project would target only poor households. For this calculation, we assume that the number of births for women in poverty are the same as the number for the rest of the women in Uganda. In 2019, there were approximately 1.65 million births in Uganda.^10^ This means that approximately 379,500 children (23 per cent * 1.65 million births) were born in households that were below the poverty line and would therefore be eligible for a nationwide voucher scheme.

Furthermore, we assume the cost of the services provided by the voucher project to be around US$60.^11^ This cost does not include the cost of surgical deliveries by caesarean section, which is approximately US$130. The total yearly cost of the of the introduction of a nationwide voucher style project would be around US$22.8 million (US$60 * 379,500 births). Our calculated infant mortality rate for non-beneficiaries is 8.2 per cent, which is a reasonable number compared with the infant mortality rate of around 3.4 per cent in Uganda considering that the focus in this study is on poor and vulnerable women. Without a voucher project, we therefore expect that around 31,100 (379,500 * 0.082) babies will not survive birth. If the voucher was to be implemented for every woman in this group, we would instead expect that 10,600 (379,500 * 0.028) babies would not survive birth. Our results from this study therefore indicate that an intervention that would give a health voucher to all poor pregnant women in Uganda has the potential to enable around 20,500 (31,000–10,600) more children to survive birth. Considering our estimated cost of such an intervention, this implies an estimated cost of around US$1,100 per child surviving birth.

## Conclusion and final remarks

The aim of this study was to measure the impact of a voucher programme in Uganda (URHVP) in which highly subsidized health care vouchers were sold to poor pregnant mothers. The first finding is that there could be high dead weight losses – mothers would have visited the health care centre even without receiving a voucher. Based on the interview answers, as many as 86 per cent of the beneficiaries said that they would have visited the health care centre regardless of the vouchers.

The second finding is that the studied voucher project shows a positive effect on the survival of babies during pregnancy and birth. The results indicate a 5% point higher probability of a child surviving if the mother was part of the voucher project than if the mother was not. It is also important to note that we compared very similar mothers when estimating this effect. The estimated effect is large and indicates that the voucher project reduced infant mortality by around 65 per cent; that is, the infant mortality in the group of beneficiaries is less than half that in the non-beneficiary group. It is also worth noting that the infant mortality rate in the group of beneficiaries (around 2.8 per cent) is lower than the infant mortality rate in Uganda as a whole: a good result of the project since the group that the voucher project targeted consisted of poor and vulnerable women who are expected to have higher infant mortality. Further, during the project implementation, there were improvements in the participating health facilities in terms of skills and management of the health workforce.

Since the voucher is found to increase the survival rate of new-borns it is positive that a new voucher scheme is being implemented to further help women with safe and facility-based deliveries. According to World Bank [29] the consolidation and scale-up of voucher projects has contributed to capacity building, institutional strengthening, and reinforcement of accountability mechanisms within the health sector in Uganda. Furthermore, they state that the implementation of voucher projects across Uganda has been known as a way of improving service delivery and health outcomes and thereby contributing to broader health care reforms. The knowledge from the different voucher schemes is important to consider when implementing new schemes or policies. According to the World Bank [29] research suggests that vouchers can act as a starting point for developing systems and expanding social health insurance.

Finally, we consider our findings from the perspective of the cost of running the programme. Based on our findings, the voucher programme has the potential to save 20,500 children’s lives at an indicative cost of US$1,100 per child surviving birth. However, we could also identify some regional heterogeneity. In the western region, babies of beneficiaries had an 8 per cent higher probability of surviving than babies of non-beneficiaries. In the eastern region, the difference in the probability of survival of a baby during pregnancy and birth between project beneficiaries and project non-beneficiaries was small and statistically insignificant. One explanation for this result might be that the western region has had more experience with health voucher systems than the eastern region, which could explain the positive results for the western region. This finding points to the importance of proper project management to achieve the greatest possible effect of a project.

Another finding is that, while conducting this evaluation, we also identified some shortcomings in the implementation of the project. One of these was that no pre-treatment data existed. We therefore recommend that the Ministry of Health in Uganda conduct baseline studies before implementation of similar projects to understand the key factors affecting project success in the different project implementation areas. These should then be incorporated into the design and implementation of the project. Collecting data for this evaluation involved around 3 months of fieldwork. We believe that allowing sufficient time and resources for evaluations of this type of project is essential. Planning for an evaluation at the start of the project could save a considerable amount of time and effort in regards to data collection. We therefore recommend that post-project evaluations are arranged during the planning and implementation stages of projects.

## Data Availability

Data is available on request to the Office of the Audit General, Kampala, Uganda.

## Footnotes

1 Health facilities were reimbursed for the transport cost of participating women to a referral facility, however there was no provision of reimbursement for return trips.

2 The districts in western Uganda were Buhweju, Kiruhura, Ibanda, Mitooma, Isingiro, Ntungamo, Kabale, Rubirizi, Kanungu, Sheema, Bushenyi and Mbarara. In eastern Uganda the districts were: Bugiri, Namutumba, Buyende, Kibuku, Kaliro, Jinja, Kamuli, Iganga, Luuka, Tororo, Mayuge, Busia, Namayingo,

3 In November 2020 Marie Stopes International changed name to MSI Reproductive Choices. MSI is an international non-governmental organization providing contraception and safe abortion services in 37 countries around the world.

4 Other methods are, for example, difference in difference see e.g. Card and Krueger, [5], which uses regression techniques. Other matching options include, for example, coarsened exact matching (CEM), introduced by Iacus et al. [10].

5 In the sensitivity analysis (Sect. 6.2), we also check whether the order of results changes if we only consider the first pregnancy during the project phase.

6 We use propensity score matching with five nearest neighbors.

7 As a sensitivity analysis we also used other matching algorithms (kernel, Mahalanobis distances and radius matching), and they all give approximately the same results regarding impacts and significance level.

8 The districts that implemented the project did not have buffer zones, however the districts are very large and serve many mothers. The risk of spillover effects is therefore deemed to be relatively low.

9 The complete table with the estimates can be obtained from the authors.

10 https://knoema.com/atlas/Uganda/topics/Demographics/Fertility/Number-of-births.

11 World Bank [27].
